# Staying hungry: a roadmap to harnessing central regulators of symbiotic nitrogen fixation under fluctuating nitrogen availability

**DOI:** 10.1007/s42994-023-00123-7

**Published:** 2023-11-18

**Authors:** Lijin Qiao, Jieshun Lin, Takuya Suzaki, Pengbo Liang

**Affiliations:** 1 State Key Laboratory of Plant Environmental Resilience, College of Biological Sciences, China Agricultural University, Beijing, China; 2 MOA Key Laboratory of Soil Microbiology, Rhizobium Research Center, China Agricultural University, Beijing, China; 3 Department of Molecular Biology and Genetics, Aarhus University, Aarhus, Denmark; 4 Faculty of Life and Environmental Sciences, University of Tsukuba, Tsukuba, Ibaraki Japan; 5 Tsukuba Plant-Innovation Research Center, University of Tsukuba, Tsukuba, Ibaraki Japan

**Keywords:** Symbiotic nitrogen fixation, N-adapted nodulation, Nodule senescence, NIN, NLPs, SNAPs

## Abstract

Legumes have evolved specific inventions to enhance nitrogen (N) acquisition by establishing symbiotic interactions with N-fixing rhizobial bacteria. Because symbiotic N fixation is energetically costly, legumes have developed sophisticated mechanisms to ensure carbon–nitrogen balance, in a variable environment, both locally and at the whole plant level, by monitoring nodule number, nodule development, and nodular nitrogenase activity, as well as controlling nodule senescence. Studies of the autoregulation of nodulation and regulation of nodulation by nodule inception (NIN) and NIN-LIKE PROTEINs (NLPs) have provided great insights into the genetic mechanisms underlying the nitrate-induced regulation of root nodulation for adapting to N availability in the rhizosphere. However, many aspects of N-induced pleiotropic regulation remain to be fully explained, such as N-triggered senescence in mature nodules. Wang et al. determined that this process is governed by a transcriptional network regulated by NAC-type transcription factors. Characterization and dissection of these soybean nitrogen-associated NAPs (SNAPs) transcription factor-mastered networks have yielded a roadmap for exploring how legumes rewire nodule functions across a range of N levels, laying the foundation for enhancing the growth of N-deprived crops in agricultural settings.

Producing enough food to feed the world’s growing population relies heavily on nitrogen (N)-containing industrial fertilizers to sustain high crop production and yields. However, the energy-intensive industrial production and use of these fertilizers account for approximately 5% of global greenhouse gas emissions (Gao and Cabrera Serrenho 2023). Additionally, soil fertility is being threatened by the overuse of fertilizers. Leguminous plants such as soybean (*Glycine max*) deploy innovations to entrap soil-borne rhizobia and allow the intracellular infection of root tissues with compatible symbionts. The recognition of specific signals from putative symbionts by the plant host triggers the development of root nodules, constituting small ecological niches in which rhizobia convert atmospheric N_2_ into ammonia, via symbiotic nitrogen fixation (SNF) (Oldroyd 2013). Thus, legumes are of ecological and economic importance for sustainable agricultural practices by bypassing the need for exogenous fertilizer application (Oldroyd and Leyser 2020).

SNF comprises an energetically costly set of biological processes within nodules whereby photosynthates are used as an energy source to drive symbiosis (Udvardi and Poole 2013; Ke et al. 2022). To balance the potential gains of N produced by symbiotic rhizobia with the carbon cost of the photosynthates these symbionts require to perform SNF, legumes have developed specialized strategies to govern symbiotic associations both systemically and locally, including monitoring nodule number, nodule development, and nodular nitrogenase activity, as well modulating nodule senescence as a function of N availability in the rhizosphere (Davidson and Robson 1986; Streeter and Wong 1988; Poole et al. 2018; Nishida and Suzaki 2018).

Autoregulation of nodulation (AON), a systemic long-range signaling pathway between roots and shoots, is responsible for maintaining an optimal number of root nodules (Fig. 1A). In soybean, the expression of *CLAVATA3/ESR*-related (*CLE*) genes, *Rhizobia-induced CLE1* (*GmRIC1*), and *GmRIC2* is induced by rhizobial infection in the roots. The encoded peptides form a root-derived signal that is perceived by the CLAVATA1-like leucine-rich repeat receptor kinase Nodule Autoregulation Receptor Kinase (GmNARK). Recognition of these peptides by GmNARK triggers the production of several shoot-derived inhibitors (SDIs) that prevent further nodule development (Reid et al. 2011; Ferguson et al. 2014). One such SDI is the phytohormone cytokinin, which moves from shoot to root to suppress the expression of the transcription factor gene *NODULE INCEPTION 1a* (*GmNIN1a*) by inducing the activity of the B-type response regulator GmRR11d (Sasaki et al. 2014; Chen et al. 2022). The perception of shoot-derived cytokinin in roots modulates the activity of the GmNIN1a–miR172c–GmNNC1 module: GmNIN1a induces the expression of *MIR172c*, whose derived mature microRNA miR172c targets the transcripts of *Nodule Number Control 1* (*GmNCC1*) for cleavage. This master switch coordinately regulates nodulation and AON, with GmNINa and GmNNC1 acting antagonistically to balance the expression of *GmRIC1* and *GmRIC2* (Wang et al. 2019) (Fig. 1A). Notably, unlike *GmRIC1* and *GmRIC2*, another CLE peptide-encoding gene, *nitrate-induced CLE 1* (*GmNIC1*), functions locally to inhibit nodulation (Reid et al. 2011).

**Fig. 1:**
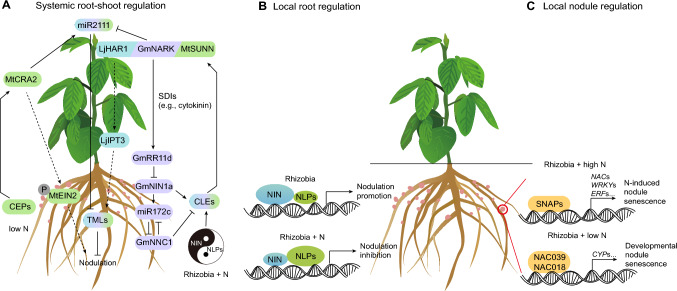
Models for the systemic and local regulation of nodulation and nodule senescence.** A** Systemic AON-mediated regulation of nodule number. Turquoise, violet, and green boxes represent known genes reported from *Lotus japonicus* (*Lj*), *Glycine ma*x (*Gm*), and *Medicago truncatula* (*Mt*), respectively. NIN and NLPs act as Yin and Yang factors to fine-tune the CLE signaling pathway, largely based on reports from *M. truncatula* and *L. japonicus*. P (in the gray circle) represents phosphorylated MtEIN2, which represses ethylene signaling locally in roots, counteracting the inhibitory effect of ethylene on nodulation. **B** NIN and NLPs in roots provide local control of nodulation in the presence of rhizobia alone (promotion) or rhizobia and high N (inhibition); the sizes of the ellipses labeled NIN and NLPs indicate their regulatory strength against downstream genes by competing for the DNA binding regions in the promoters of downstream genes. **C** Local nodule senescence regulation is mastered by the SNAP-centered pathway under high N; the developmental senescence of nodules is controlled by the NAC-type transcription factors NAC039 and NAC018

This negative feedback regulatory pathway is conserved across legumes such as *Lotus japonicus* and *Medicago truncatula* (Schnabel et al. 2005; Mortier et al. 2010, 2012; Nishida et al. 2016; Hastwell et al. 2017; Gautrat et al. 2019; Zhang et al. 2021). This observation suggests that other model legumes might share the complete feedback signaling circuit present in soybean, and vice versa. In *L. japonicus*, miR2111 undergoes leaf-to-root translocation to control symbiosis through posttranscriptional cleavage of transcripts for the symbiosis suppressor gene *TOO MUCH LOVE* (*TML*) in roots (Tsikou et al. 2018; Okuma et al. 2020). In *M. truncatula*, this leaf-to-root translocation relies on another leucine-rich repeat receptor kinase, compact root architecture 2 (CRA2), a receptor for root-derived C-terminally encoded peptide (CEP)-type peptides, maintaining a high level of *MIR2111* expression in non-inoculated plants to promote the competence for nodulation (Gautrat et al. 2020). Importantly, AON-related mutants generally exhibit nodulation even under high-nitrate conditions (nitrate tolerance), a condition that commonly suppresses nodulation in wild-type plants (Nishida et al. 2020).

Additionally, ethylene negatively regulates symbiosis, as the external application of ethylene inhibits nodulation (Oldroyd et al. 2001). Mutants of the ethylene signaling pathway component EIN2 (ethylene insensitive 2) in *M. truncatula* display a hypernodulation phenotype (Penmetsa and Cook 1997). Studies from alfalfa (*Medicago sativa*) showed that nitrate treatment increases ethylene content in roots, whereas treatment with aminoethoxyvinylglycine (AVG), an inhibitor of ethylene biosynthesis, improves the nitrate tolerance of nodulation (Ligero et al. 1991). These findings suggest that ethylene plays a role in N-mediated inhibition of nodulation. Notably, under N-limited conditions, the MtCEP1–MtCRA2 pathway balances root and nodule development by reducing auxin biosynthesis and repressing ethylene signaling (Zhu et al. 2020). Hence, the integration of phytohormones and their corresponding signaling pathways into AON provides a more comprehensive understanding of this systemic signaling circuit dedicated to controlling nodulation (Fig. 1A).

Apart from systemic AON regulation, NIN and NLPs mediate the local inhibition of nodulation by high N status in roots (Fig. 1B). MtNLP1 is involved in the nitrate signaling pathway, with nitrate triggering the shuttling of MtNLP1 from the cytosol to the nucleus where it suppresses the NIN-mediated transcriptional activation of target symbiotic genes (Lin et al. 2018). Similar NLP-mediated, nitrate-induced control of root nodule symbiosis has been uncovered in *L. japonicus*, as proteins related to LjNIN and LjNLP1 likely competitively bind to *cis*-elements present in the promoters of downstream genes, such as the nitrate transporter gene *LjNRT2.1* (Nishida et al. 2018, 2021; Misawa et al. 2022). Moreover, high nitrate levels restrict nodule organogenesis by mediating the expression of *Isopentenyl transferase 3* (*LjIPT3*), whose encoded protein participates in cytokinin biosynthesis, in an LjNLP1- and LjNLP4-dependent manner (Sasaki, et al. 2014; Lin et al. 2021). Notably, recent evidence indicates crosstalk between AON and NLPs or NIN (Nishida et al. 2018; Laffont et al. 2020; Luo et al. 2021), suggesting that a finely tuned regulatory loop is required to maintain systemic root–shoot and local root regulation of nodule number (Fig. 1A, B).

High soil nitrate is known to suppress nodule development and nodular nitrogenase activity. Different hypotheses have been proposed to explain how high N inhibits nodular nitrogenase activity, including feedback inhibition by a product of nitrate metabolism such as glutamine or asparagine (Bacanamwo and Harper 1997; Neo and Layzell 1997), carbon starvation (Vessey et al. 1988; Streeter and Wong 1988; Thorpe et al. 1998; Fujikake et al. 2003), and restriction of bacteroid respiration caused by the insufficient diffusion of O_2_ into nodules (Schuller et al. 1988; Vessey et al. 1988; Gordon et al. 2002). High soil nitrate levels also accelerate nodule senescence, eventually leading to nodule disintegration. This physiological and developmental alteration is reflected by a visible change in nodule color from red to green, as functional nodules filled with O_2_-bound leghemoglobin undergo leghemoglobin nitration and breakdown, resulting in cessation of the nitrogenase activity for SNF (Dupont et al. 2012). However, the molecular mechanisms and signaling networks involved in this switch are still poorly understood. Legume nodule senescence might be regulated by a gene regulatory network that is distinct from previously characterized N-responsive networks, as nitrate generally promotes plant growth rather than senescence (O'Brien et al. 2016).

Wang et al. harnessed the power of a hydroponic culture system to follow the physiological and developmental changes in mature nodules in a spatiotemporal manner through the rapid and reversible treatment of soybean roots with various N conditions (Wang et al. 2023b). The authors performed a top–down analysis of 13,446 differentially expressed genes identified through transcriptome deep sequencing (RNA-seq) analysis by constructing a co-expression network in soybean nodules. Before assembling this network, the authors thoroughly investigated the cell morphology of N-induced nodule senescence, by exploring the visible shift in nodule color and decreased leghemoglobin content and using high-resolution imaging to examine vacuolar morphology and the accumulation of poly-β-hydroxybutyrate (PHB) polymers in bacteroides (Wang et al. 2023b).

Assembly of the co-expression network revealed a dynamic and reversible transcriptional network underlying the inhibition of N_2_ fixation by high N (10 mM total N, 8 mM nitrate + 2 mM ammonium) conditions. Among the most highly connected transcription factors were a group of NAC-type transcription factors known as soybean nitrogen-associated NAPs (SNAPs).

To explore the genes associated with the modulation of SNF, under a range of N conditions, Wang et al. also assembled a weighted gene co-expression network by combining RNA-seq data and results from an acetylene reduction assay (ARA) as a phenotypic output to estimate potential nitrogenase activity. The authors focused on two modules that showed opposite correlations between the expression levels of their constituent genes and ARA activity, pointing to their association with the control of nodulation. The genes whose expression levels were most positively correlated with ARA activity were enriched for the gene ontology (GO) terms “microtubule-based movement”, “spindle organization”, and “cellular amino acid biosynthetic process”. Genes with an expression pattern opposite that of ARA activity showed enrichment for the GO terms “trehalose biosynthetic process”, “autophagic vacuole assembly”, and “regulation of transcription”. This contrasting type of regulation is in line with symptoms of senescence. Indeed, previous studies have shown that trehalose abundance and autophagic activity increase during nodule senescence (Müller et al. 2001; Vauclare et al. 2010), while fast cytoskeleton-based movement and cellular amino acid biosynthesis suggest an increasingly active cellular response in nodules (Björkeroth et al. 2020; McKenney et al. 2014). More effort will be required to unravel the detailed mechanisms in follow-up studies.

Enrichment for the GO term “regulation of transcription” among the N-induced modules suggested the crucial roles of transcription factors in N-induced transcriptome reprogramming. Notably, six of the top eight most connected transcription factors in this module belong to the SNAC-B/NAP sub-family, suggesting that members of this subfamily might be crucial for the N-induced transcriptional response. The entire subfamily was designated as *SNAP1–11*. Two *snap1/2/3/4* quadruple mutants were generated, via CRISPR/Cas9-mediated mutagenesis, to dissect their functions genetically. The *snap1/2/3/4* mutants exhibited normal vegetative growth, nodule number, and nitrogenase activity when grown under low N conditions. Importantly, however, the quadruple mutants behaved very differently under high N supply. ARA activity and red leghemoglobin content were higher in the mutants than the wild type; the *snap1/2/3/4* mutants also showed fewer alterations in symbiosome formation and smaller lytic vacuolar compartments than the wild type.

However, we might lack an integrated view of SNAP function in the context of N-induced inhibition of nodule activity and N-induced senescence, as *SNAP1*, *SNAP2*, and *SNAP4* are not highly expressed in nodules under high N supply, unlike *SNAP9*, *SNAP8*, and *SNAP10*. That said, we cannot exclude the possibility that SNAP members exhibit partial functional redundancy, despite the high expression of their encoding genes is not necessarily a prerequisite for their shared function. The fact that SNAP transcription factors can cross activate the expression of other *SNAP*s might partially explain this result. For example, SNAP1, SNAP2, and SNAP3 all target *SNAP8*, whereas SNAP1 targets *SNAP2* and *SNAP10* (Wang et al. 2023b). Disentangling the functional differences among SNAPs might help identify their specific and shared downstream target genes.

To explore the genes directly or indirectly targeted by the above SNAPs during N-induced senescence, Wang et al. performed chromatin immunoprecipitation followed by sequencing (ChIP-seq) of the wild type and the *snap1/2/3/4* quadruple mutants and combined these results with RNA-seq data. Notably, the SNAPs directly activate the expression of *NAC*, *ethylene-response factor* (*ERF*), and *WRKY* transcription factor genes (Fig. 1C). In agreement with this finding, these genes were induced in the wild type but to a lesser extent in *snap1/2/3/4* nodules under high N conditions. This finding aligns with the function of NAC transcription factors in promoting plant senescence and the alleviation of N-induced nodule senescence in the *snap1/2/3/4* mutants. This NAC-centered network and related datasets pave the way for a detailed dissection of the mechanisms mediated by different downstream transcription factors and advance our understanding of N-induced nodule senescence.

One question comes to the forefront: do the genes downstream of SNAPs mainly regulate nodules locally or do they contribute to signaling modules in other tissues that affect diverse processes, such as leaf senescence? For example, the downstream gene *Vacuolar Processing Enzyme* (*GmVPE*) is expressed in soybean cotyledons and leaves in addition to roots (Liu et al. 2023). Moreover, *GmWRKY22* is expressed at higher levels in roots than in nodules according to published transcriptome data (Adhikari et al. 2019; Liu et al. 2023). Thus, these local SNAP-mediated nodule networks may bring new insights into other tissue-dependent mechanisms, such as leaf senescence. Combining these SNAP-centered networks with evolutionary analysis of SNAPs and their downstream genes among legumes and non-legumes could potentially help elucidate the functional specificity of SNAPs: do they function specifically in SNF or globally in multiple pathways and/or tissues?

It would also be interesting to investigate whether SNAP-centered networks are connected to the pathways that control AON or to pathways controlling nodule number in a NIN- and NLPs-dependent manner, either in space or time. For instance, does nitrate availability affect the translocation of the transcription factor NAC075 for cell-type-specific reprogramming of root growth, as shown in non-legume plant species (Xiao et al. 2022)? Intriguingly, the nodule senescence-related transcription factor LjNAC094 was recently shown to function downstream of LjNLP1 and LjNLP4 (Wang et al. 2023a). In an independent study, GmNAC039 and GmNAC018 were demonstrated to promote developmental senescence of soybean nodules by activating the expression of cysteine protease (*CYP*s) genes under relatively low levels of nitrate supplementation (0.5 mM nitrate, Yu et al. 2023). These findings strongly support the idea that NAC-centered networks play a major role in nodule senescence, with the caveat that NACs likely play diverse functions in response to different environmental cues (Fig. 1C).

Nitrate and ammonium are the preferred N forms used by plants (O'Brien et al. 2016), but the effects of different N forms on plant growth and nodulation should be carefully examined in all experiments, as N can also be present in the form of urea and glutamine (Yamashita et al. 2019). The study by Wang et al. (2023b) used a mixture of nitrate and ammonium at a 4:1 ratio, with 10 mM total N. The regulatory network uncovered by this study may thus be distinct from that identified as triggering senescence using pure nitrate or pure ammonium as a single N source. For example, the application of nitrate only was predominantly used in a body of work focused on N-induced responses in model legumes, with nitrate representing N (Lin et al. 2021, 2018; Luo et al. 2021; Misawa et al. 2022; Nishida et al. 2018). Of note, ammonium, but not nitrate, attenuates nodulation in *nlp4* mutants, suggesting that NLPs are not required for the ammonium response (Nishida et al. 2018). In general, plants use different transporters for N uptake, which might result in differences in efficiency and priority under conditions with various N sources (O'Brien et al. 2016). Hence, uncovering the nodulation responses to these N sources are important next questions in the field.

Besides N-induced senescence, senescence can also be induced by stressful environmental conditions, such as prolonged exposure to darkness in shoots, which triggers rapid, global nodule senescence (Pérez Guerra et al. 2010). If such nodule senescence is caused by nutrient remobilization from sink to source, a distinct and more responsive signaling pathway must operate, which remains to be identified. Is this type of nodule senescence triggered by light-induced mobile factors? It would be interesting to evaluate N-induced senescence in light-related soybean mutants whose nodulation is aborted, such as *soybean TGACG-motif-binding Factor 1* (*stf1*), *stf2*, *stf3*, *stf4, flowering locus t2a* (*ft2a*), and *elongated hypocotyl 5* (*hy5*) (Wang et al. 2021; Ji et al. 2022). Of course, how plants growing in the field respond to different abiotic and biotic stress conditions, in general and in terms of nodule senescence, remains a major open question.

In conclusion, the study by Wang et al. (2023b) pioneered the use of a top–down strategy to construct a SNAP-centered expression network and explore N-induced senescence. Their findings bring us one step closer to optimizing and creating ideal soybean varieties with N-insensitive SNF that are better adapted to the fluctuating environments in agricultural settings.

## Data Availability

Data sharing is not applicable to this article as no datasets were generated or analyzed during the current study.
